# Self-reported symptoms and healthcare seeking in the general population -*exploring “The Symptom Iceberg”*

**DOI:** 10.1186/s12889-015-2034-5

**Published:** 2015-07-21

**Authors:** Sandra Elnegaard, Rikke Sand Andersen, Anette Fischer Pedersen, Pia Veldt Larsen, Jens Søndergaard, Sanne Rasmussen, Kirubakaran Balasubramaniam, Rikke Pilsgaard Svendsen, Peter Vedsted, Dorte Ejg Jarbøl

**Affiliations:** Research Unit of General Practice, Department of Public Health, University of Southern Denmark, J.B. Winsløws Vej 9A, 5000 Odense C, Denmark; The Research Unit for General Practice, Research Centre for Cancer Diagnosis in Primary Care - CaP, Department of Public Health, Aarhus University, Bartholins Allé 2, 8000 Aarhus C, Denmark

**Keywords:** General practice, Symptom experience, Questionnaire, Healthcare seeking, Gender, Symptom iceberg, Population based, Denmark

## Abstract

**Background:**

Research has illustrated that the decision-making process regarding healthcare seeking for symptoms is complex and associated with a variety of factors, including gender differences. Enhanced understanding of the frequency of symptoms and the healthcare seeking behaviour in the general population may increase our knowledge of this complex field.

The primary objective of this study was to estimate the prevalence of self-reported symptoms and the proportion of individuals reporting GP contact, in a large Danish nationwide cohort. A secondary objective was to explore gender differences in GP contacts in response to experiencing one of the 44 predefined symptoms.

**Methods:**

A Danish nationwide cohort study including a random sample of 100,000 individuals, representative of the adult Danish population aged 20 years or above. A web-based questionnaire survey formed the basis of this study. A total of 44 different symptoms covering a wide area of alarm symptoms and non-specific frequently occurring symptoms were selected based on extensive literature search. Further, items regarding contact to the GP were included. Data on socioeconomic factors were obtained from Statistics Denmark.

**Results:**

A total of 49,706 subjects completed the questionnaire. Prevalence estimates of symptoms varied from 49.4 % (24,537) reporting tiredness to 0.11 % (54) reporting blood in vomit. The mean number of reported symptoms was 5.4 (men 4.8; women 6.0).

The proportion of contact to the GP with at least one symptom was 37 %. The largest proportion of GP contacts was seen for individuals reporting blood in the urine (73.2 %), whereas only 11.4 % of individuals with increase in waist circumference reported GP contact. For almost 2/3 of the symptoms reported, no gender differences were found concerning the proportion leading to GP contacts.

**Conclusion:**

Prevalence of symptoms and GP contacts are common in this overview of 44 different self-reported symptoms. For almost 2/3 of the reported symptoms no gender differences were found concerning the proportion leading to GP contacts. An enhanced understanding of healthcare seeking decisions may assist healthcare professionals in identifying patients who are at risk of postponing contact to the GP and may help development of health campaigns targeting these individuals.

## Background

Knowledge about symptoms and healthcare seeking decisions provides an arena for understanding the interface between the healthcare system and the population. Since the 1960s we have witnessed a series of studies exploring the prevalence of symptoms and the proportion of healthcare seeking [1–5]. This phenomenon was identified as “The Symptom Iceberg” for the first time in 1963 by JM Last [1] and operationally defined by Hannay in 1979 [6]. The phenomenon depicts two parts – the “submerged part” encompassing the majority of symptom experiences, which are not brought to the attention of a general practitioner (GP), and the “surfaced part” symbolising the proportion of symptoms, which are presented to the GP.

The prevalence of self-reported symptoms varies in the existing literature. Two recent studies estimated the prevalence of symptoms, but in two different settings: a community-based survey among people with musculoskeletal complaints explored the prevalence of 25 different symptoms [7] and a population based study drawn from general practices in the UK explored the prevalence of 23 different symptoms [8]. They found an average number of symptoms experienced during the preceding 2 weeks of 3.7 and 6.0, respectively [7, 8]. Further, research has found a wide range in the proportion who contacted the GP in response to a symptom, from 5–25 % [7–14].

Studies have illustrated that the decision process regarding healthcare seeking for a symptom is complex and depends on a variety of different factors, which possibly differ among men and women [15]. It has been argued that women are socialised to pay more attention to their bodies and tend to seek more medical advice than men [16]. However, the greater tendency to consult amongst women is not consistent in the literature [15, 17].

From a public health perspective people’s decision about healthcare seeking is important with regard to improvements in risk profiling and diagnostics, such as e.g. cancer diagnostics. Symptoms potentially indicative of serious disease should *preferably* lead to healthcare seeking, while other symptoms should not. However, it is a challenge that most symptoms have low positive predictive values for serious disease [18]. Further, the awareness that some symptoms may be a sign of serious disease may differ among different groups in the population [19]. This has to be systematically explored in large-scale studies in a general population. An enhanced understanding of the size of the pool of symptoms and subsequent consequences in the population may improve policy interventions targeting healthcare seeking, e.g. systematic patient delays. Investigating a wide range of self-reported symptoms and the subsequent healthcare seeking decision is therefore important.

The primary objective of this study was to estimate the prevalence of self-reported symptoms and the proportion of individuals reporting GP contact, in a large Danish nationwide cohort. A secondary objective was to explore gender differences in GP contacts in response to experiencing one of the 44 predefined symptoms.

## Methods

### Study design

This study was part of a Danish nationwide cohort comprising a random sample of 100,000 individuals, representative of the adult Danish population aged 20 years or above. The overall aim of the cohort study was to estimate the prevalence of symptoms among individuals in the general population, the individuals’ interpretation of symptoms, related factors influencing the decision to contact the GP and their healthcare-seeking behaviour. Further, the cohort will be followed-up using registers on health care utilization and hospital admissions to explore the predictive values of the symptoms for various diseases.

Baseline data presented in this paper were collected in a web-based survey. The data collection was conducted from June to December 2012, thereby excluding the months where the flu activity in Denmark normally peaks.

### Subjects and sampling

All Danish citizens are registered with a unique personal identification number in the Danish Civil Registration System (CRS), which contains information on any Danish resident’s date of birth, gender, migration, etc. The CRS enables accurate linkage between all national registers [20]. The sample for this study was randomly selected using the CRS and was invited to participate in the survey. Each individual received a postal letter explaining the purpose of the study. In the letter a unique 12-digit login for a secure webpage was included. This provided access to a comprehensive web-based questionnaire [21].

The initial invitation letter was followed by a reminder to non-respondents after two weeks. After an additional two weeks the non-respondents were contacted by telephone and encouraged to participate. In order to prevent the exclusion of people with no access to a computer, tablet or smartphone, the participants were offered the opportunity to respond to the survey in a telephone interview. Information on severe illness and subjects who had moved abroad was occasionally provided by family or relatives in the reminder procedure [21].

### Questionnaire

A comprehensive questionnaire including 44 different symptoms covering a wide area of clinically relevant predefined symptoms was developed. For representativeness of symptoms that from a medical perspective are defined as indicating a serious disease, we selected a number of alarm symptoms of cancer covering the following areas: lung, gastrointestinal, gynaecological, and urogenital cancer. These items were selected based on a review of literature, national and international cancer referral guidelines and descriptions of cancer pathways [22–24]. In addition, we included a number of frequently occurring symptoms, which are often presented to the GP, e.g. back pain, headache and tiredness. Items regarding each specific symptom were phrased: “Have you experienced any of the following bodily sensations, symptoms or discomfort within the past four weeks?” With regard to GP contact, the question was worded for each selected symptom: “Have you contacted your general practitioner concerning the symptom(s) you have experienced within the past four weeks, through appointment, by telephone or e-mail?”

The questionnaire was pilot- and field-tested and adjusted in light of the results from these. The methodological framework for developing the questionnaire is described in details elsewhere [21].

### Responder analysis

In order to compare the study sample, respondents and non-respondents, data on socioeconomic and demographic factors were collected from Statistics Denmark [25]. For each individual we obtained information on education, income, labour market affiliation, cohabitation status, ethnicity and average number of contacts to the GP. Information was retrieved for the year 2011, i.e. the year preceding the questionnaire study. Education was categorised according to the length of the highest attained educational level: low (<10 years (primary and lower secondary school)); middle (10–12 years (vocational education and upper secondary school)); and high (>12 years (short-, medium- and long-term higher education)). This categorisation was selected to reflect the organisation of the Danish educational system [26]. Equivalence weighted disposable income was categorised as low income (1st quartile), middle income (2nd and 3rd quartile), and high income (4th quartile). Labour market affiliation was categorised into three groups: (i) working, (ii) pensioners and (iii) out of the workforce. Cohabitation status was categorised into: cohabiting/married or single. Ethnicity was categorised into three groups: persons with Danish origin, immigrants, and descendants of immigrants. The total number of contacts to the GP in 2011 was obtained from the National Health Service Register [25].

### Statistical analysis

The following socioeconomic and demographic characteristics of the study sample, respondents and non-respondents were described: sex, age, education, income, labour market affiliation, cohabitation status, ethnicity and average contacts to GP the preceding year. Chi-square tests were used to test for differences between characteristics of respondents and non-respondents.

Prevalence estimates of each reported symptom and the proportion of individuals with contact to the GP were calculated with 95 % confidence intervals based on the binominal distribution. The reported symptoms were ranked according to their frequency. Respondents answering ”not relevant for me” were excluded from the analysis and the answers “do not wish to answer” which accounted for less than 5 %, was considered as missing and not included in the analyses. In order to explore the pattern of “The Symptom Iceberg” for each gender, the prevalence of symptom experiences and proportion of contacts to the GP were stratified on gender. We tested whether the prevalence estimates differed between genders using chi-squared tests. Contacts to GP were ranked separately for men and women, according to the proportion contacting the GP in response to experiencing a symptom.

A histogram of the number of reported symptoms by the participants was constructed for the full sample as well as for men and women separately. For each number of symptoms, the proportion contacting the GP with at least one of the symptoms was indicated. All data analyses were conducted using StataIC 13©.

### Ethical approval

The Regional Scientific Ethics Committee for Southern Denmark evaluated the project and concluded that no further approval was necessary due to Danish legislation. The participants in the study were clearly informed that there would be no clinical follow-up, and that they should contact their own GP in case of concern or worry. The project was approved by the Danish Data Protection Agency (journal no. 2011-41-6651).

## Results and discussion

**Fig. 1:**
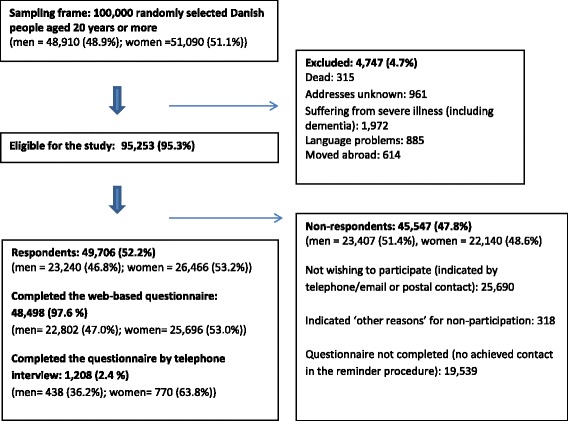
Study cohort

Of the 100,000 randomly selected subjects, 4,474 (4.7 %) were not eligible because they had either died, were suffering from severe illnesses (including dementia), had language problems, had moved abroad or could not be reached due to unknown address. Of the 95,253 (95.3 %) eligible subjects, 49,706 subjects completed the questionnaire, yielding a response rate of 52.2 %. Some 1,208 (2.4 %) completed the questionnaire by telephone. Of all non-respondents, 26,008 (57.1 %) indicated that they did not wish to participate in the study, whereas for the remaining 19,539 (42.9 %) no contact was achieved during the reminder procedure (Fig. 1). The electronic format of the questionnaire enabled a leap structure, so the respondents were directed through the questionnaire according to their previously given answers, skipping irrelevant questions. Further, the structure ensured that respondents were required to answer each question in order to continue to the next. Less than 5 % of the respondents did not complete the questionnaire.

Table 1 shows socioeconomic and demographic characteristics of the total study sample, respondents and non-respondents, respectively. The median age of the study sample was 51 years (IQR 38–65). Median age of respondents was slightly higher than non-respondents; 52 years (IQR 40–64) compared to 50 years (IQR 36–66), respectively. The respondents were fairly representative of the study sample. However, more respondents were females, married/living together, had a high educational and income level and were attached to the labour market. Differences between respondents, non-respondents and the study sample according to descriptive characteristics are shown in Table 1.

**Table 1:** Descriptive characteristics of the total sample, respondents and non-respondents in the survey (N = 100 000)

	Total sample	Respondents	Non-respondents				
	N	%	n	%	n	%	P-value*
Sex							
*Male*	48 910	48.9	23 240	46.8	23 407	51.4	<0.001
*Female*	51 090	51.1	26 466	53.2	22 140	48.6	
Age							
*20*–*39*	27 706	27.7	12 251	24.6	15 455	30.7	<0.001
*40*–*59*	37 106	37.1	20 305	40.9	16 801	33.4	
*60*–*79*	28 868	28.9	15 748	31.7	13 120	26.1	
*80-*	6 320	6.3	1 402	2.8	4 918	9.8	
Marital status^a^							
*Single*	31 140	32.8	12 475	25.1	18 665	41.2	<0.001
*Married/living together*	63 807	67.2	37 140	74.9	26 667	58.8	
Educational level^a^							
*Low*	24 770	27.2	9 540	19.7	15 230	35.6	<0.001
*Medium*	40 659	44.6	22 155	45.8	18 504	43.3	
*High*	25 752	28.2	16 724	34.5	9 028	21.1	
Income level^a^							
*Low*	22 440	23.6	8 072	16.3	14 368	31.7	<0.001
*Medium*	48 126	50.7	25 712	41.8	22 414	49.4	
*High*	24 382	25.7	24 382	31.9	8 551	18.9	
Employment status^a^							
*Workning*	59 961	63.1	33 961	68.4	26 000	57.3	<0.001
*Pensioners*	23 193	24.4	11 294	22.7	11 899	26.2	
*Out of workforce*	11 911	12.5	4 410	8.9	7 501	16.5	
Ethnic groups^a^							
*Danish*	86 248	90.8	46 543	93.8	39 705	87.6	<0.001
*Immigrants*	8 038	8.5	2 858	5.8	5 180	11.4	
*Descendants of Immigrants*	661	0.7	214	0.4	447	1.0	
GP contacts^a^							
*Average contacts to GP in 2011*	8.1		7.6		8.5		<0.001

^a^ *Total numbers for each group may not add up to full sample, 5 to 9* % *missing data from Statistics Denmark*

*Differences between respondents and non-respondents according to descriptive characteristics were tested using chi-square tests

Prevalence estimates of self-reported symptoms in the preceding four weeks and the proportions of individuals with report of contact to the GP are listed in Table 2. Prevalence estimates of symptoms varied from 49.4 % (24,537) reporting tiredness to 0.11 % (54) reporting blood in vomit. The symptoms are ranked by frequency. The largest proportion of GP contacts was observed for individuals reporting blood in the urine 73.2 %, whereas 11.4 % of individuals with increase in waist circumference reported contact to the GP (Table 2).

**Table 2:** *The Symptom Iceberg* – Prevalence of self-reported symptoms in the previous 4 weeks and the proportion of GP contacts. Ranked from 1 to 44 according to proportion of symptoms in the study population

	Proportion with symptoms				Proportion with GP contacts		
	N	%	[95 % CI]	Rank	N	%	[95 % CI]
Tiredness	24 537	49.8	[49.4–50.3]	1	4 907	20.2	[19.7–20.7]
Night-time urination	23 935	48.7	[48.2–49.1]	2	3 024	12.8	[12.3–13.2]
Lack of energy	18 472	37.5	[37.1–37.9]	3	3 599	19.7	[19.1–20.3]
Headache	17 978	36.5	[36.1–37.0]	4	3 159	17.7	[17.2–18.3]
Back pain	15 925	32.3	[31.9–32.8]	5	5 490	34.9	[34.1–35.6]
Abdominal bloating	14 712	29.8	[29.4–30.2]	6	1 864	12.9	[12.3–13.4]
Memory problems	9 824	19.9	[19.6–20.3]	7	1 771	18.3	[17.6–19.1]
Abdominal pain	9 765	19.6	[19.4–20.1]	8	2 659	27.8	[26.9–28.7]
Erectile dysfunction^a^	4 289	19.3	[18.8–19.8]	9	1 362	32.1	[30.7–33.5]
Coughing	8 804	17.9	[17.5–18.2]	10	2 120	24.4	[23.5–25.3]
Concentration problems	8 662	17.6	[17.2–17.9]	11	1 742	20.4	[19.6–21.3]
Change in stool texture	8 543	17.3	[17.0–17.6]	12	1 260	15.0	[14.3–15.8]
Dizziness	7 889	16.0	[15-7-16-3]	13	2 407	30.9	[29.9–32.0]
Pelvic pain^a^	3 963	15.4	[14.9–15.8]	14	1 008	25.8	[24.4–27.2]
Feeling unwell	7 411	15.0	[14.7–15.4]	15	2 065	28.3	[27.3–29.3]
Constipation	7 231	14.7	[14.3–15.0]	16	970	13.6	[12.9–14.5]
Increase in waist circumference	6 548	13.3	[13.0–13.7]	17	733	11.4	[10.6–12.2]
Change in stool frequency	6 466	13.1	[12.8–13.4]	18	1 009	15.9	[15.0–16.8]
Diarrhoea	6 385	12.9	[12.7–13.2]	19	1 057	16.8	[15.9–17.7]
Nausea	6 256	12.6	[12.3–12.9]	20	1 264	20.6	[19.6–21.6]
Swollen legs	6 056	12.3	[12.0–12.6]	21	2 224	37.2	[36.0–38.5]
Difficulty in emptying the bladder	5 731	11.6	[11.4–11.9]	22	1 534	27.1	[26.0–28.3]
Frequent urination	5 234	10.6	[10.4–10.9]	23	1 362	26.5	[25.3–27.7]
Pelvic pain during intercourse^a^	2 091	10.2	[9.8–10.6]	24	552	26.6	[24.8–28.6]
Stress incontinence	4 797	9.8	[9.5–10.0]	25	852	18.0	[16.8–19.1]
Shortness of breath	3 960	8.0	[7.8–8.3]	26	1 936	49.7	[48.1–51.2]
Hoarseness	3 782	7.7	[7.4–7.9]	27	698	18.7	[17.5–20.0]
Urge incontinence	3 080	6.3	[6.0–6.5]	28	790	26.1	[24.5–27.6]
Loss of appetite	3 079	6.3	[6.0–6.5]	29	586	19.4	[18.1–20.9]
Blood in stool/rectal bleeding	2 285	4.6	[4.4–4.8]	30	758	33.7	[31.8–35.7]
Fever	1 952	4.0	[3.8–4.1]	31	517	26.8	[24.9–28.8]
Difficulty swallowing	1 727	3.5	[3.3–3.7]	32	586	34.9	[32.6–37.2]
Weight loss	1 490	3.0	[2.9–3.2]	33	363	25.1	[23.0–27.4]
Vaginal bleeding after intercourse^a^	612	3.0	[2.8–3.2]	34	187	30.9	[27.2–34.7]
Incontinence without stress/urge	1 158	2.3	[2.2–2.5]	35	383	33.8	[31.1–36.6]
Postmenopausal bleeding^a^	370	2.3	[2.1–2.5]	36	118	33.1	[28.2–38.2]
Pain/burning when urinating	1 046	2.1	[2.0–2.3]	37	489	47.8	[44.7–50.8]
Lump/swollen lymph nodes	811	1.6	[1.5–1.8]	38	332	41.5	[38.1–45.0]
Black stool	779	1.6	[1.5–1.7]	39	132	17.3	[14.8–20.2]
Repeated vomiting	643	1.3	[1.2–1.4]	40	208	33.6	[30.0–37.4]
Blood in urine	284	0.6	[0.5–0.7]	41	202	73.2	[67.6–78.1]
Blood in semen^a^	94	0.4	[0.3–0.5]	42	45	48.9	[38.7–59.2]
Coughing up blood	62	0.1	[0.1–0.2]	43	29	47.5	[35.1–60.3]
Blood in vomit	54	0.1	[0.1–0.1]	44	17	37.0	[23.9–52.2]

^a^ *Gender specific symptoms*

**Fig. 2:**
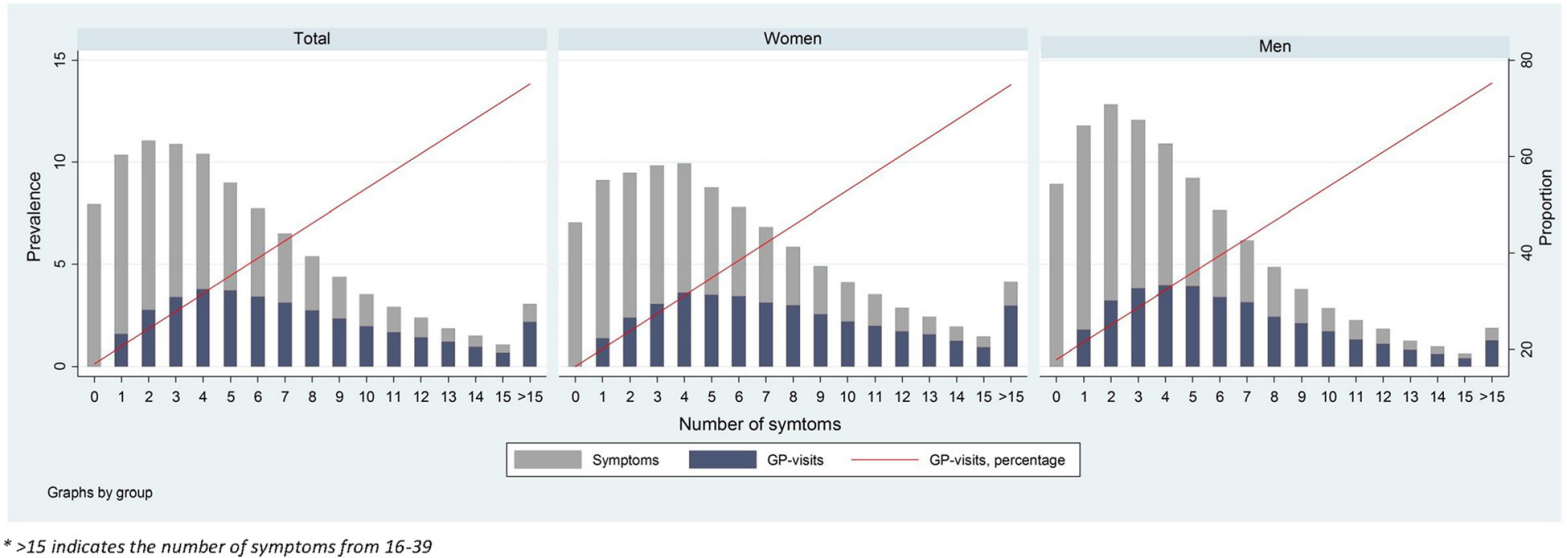
The graphs show the proportion who experienced the given number of symptoms (light blue bar) and the proportion with the given number of symptoms who contacted the GP with at least one symptom (dark blue bar). The red line and the right y-axis refer to the linear relationship between the number of symptoms and the proportion of GP contacts among individuals with the given number of symptoms. The graph is shown for the total sample and for men and women separately

About 9 out of 10 respondents reported at least one symptom within the preceding four weeks. The mean number of reported symptoms was 5.4 (men: 4.8; women: 6.0, p < 0.001). The number of symptoms reported ranged from 0 to 39. Figure 2, illustrates the proportion who reported the given number of symptoms and the proportion with the given number of symptoms who contacted the GP with at least one symptom. Women were most likely to have reported four symptoms within the preceding four weeks, while men were most likely to have reported two symptoms. The proportion of symptoms leading to GP contacts increased with increasing number of symptoms experienced. This was similar for both men and women (Fig. 2). The gender-specific prevalences of reported symptoms and proportions of GP contact are listed in Table 3.

**Table 3:** Prevalence of symptoms and GP contacts, stratified on gender. Proportions of GP-contacts were ranked from 1 to 42 according to frequency

	Proportion with symptoms			Proportion with GP contacts				
	*Gender*	*n*	*%*	*n*	*%*	*[95* % *CI]*	*Rank*	p-value*
Tiredness	Men	10 642	45.8	1 923	18.3	[17.5–19.0]	28	<0.001
	Women	13 895	52.5	2 984	21.7	[21.0–22.4]	26	
Night-time urination	Men	11 424	49.2	1 928	17.0	[16.3–17.7]	31	<0.001
	Women	12 511	47.3	1 096	8.9	[8.4–9.4]	42	
Lack of energy	Men	8 215	35.3	1 437	17.7	[16.8–18.5]	29	<0.001
	Women	10 257	38.8	2 162	21.4	[20.6–22.2]	27	
Headache	Men	6 675	28.7	1 016	15.3	[14.5–16.2]	36	<0.001
	Women	11 303	42.7	2 143	19.2	[18.4–19.9]	34	
Back pain	Men	7 067	30.4	2 468	35.2	[34.1–36.3]	10	0.437
	Women	8 858	33.5	3 022	34.6	[33.6–35.6]	8	
Abdominal bloating	Men	5 073	21.8	674	13.5	[21.5–14.5]	38	0.101
	Women	9 639	36.4	1 190	12.5	[11.9–13.2]	40	
Memory problems	Men	4 177	18.0	691	16.8	[15.7–18.0]	32	0.001
	Women	5 647	21.3	1 080	19.5	[18.4–20.5]	33	
Abdominal pain	Men	3 273	14.1	1 002	31.3	[29.7–32.9]	16	<0.001
	Women	6 492	24.5	1 657	26.0	[25.0–27.2]	19	
Coughing	Men	4 212	18.1	953	22.9	[21.6–24.2]	24	0.002
	Women	4 592	17.4	1 167	25.7	[24.5–27.0]	21	
Concentration problems	Men	3 566	15.3	687	19.5	[18.2–20.9]	26	0.089
	Women	5 096	19.3	1 055	21.0	[19.9–22.2]	28	
Change in stool texture	Men	3 858	16.6	542	14.3	[13.2–15.4]	37	0.083
	Women	4 685	17.7	718	15.6	[14.6–16.7]	37	
Dizziness	Men	3 101	13.3	961	31.3	[29.7–33.0]	15	0.557
	Women	4 788	18.1	1 446	30.7	[29.4–32.0]	14	
Feeling unwell	Men	3 042	13.1	831	27.7	[26.1–29.3]	19	0.337
	Women	4 369	16.5	1 234	28.7	[27.4–30.1]	15	
Constipation	Men	2 422	10.4	317	13.3	[12.0–14.7]	39	0.542
	Women	4 809	18.2	653	13.8	[12.9–14.8]	39	
Increase in waist circumference	Men	2 266	9.7	217	9.7	[8.5–11.0]	40	0.002
	Women	4 282	16.2	516	12.3	[11.3–13.3]	41	
Change in stool frequency	Men	2 757	11.9	444	16.5	[15.1–17.9]	33	0.308
	Women	3 709	14.0	565	15.5	[14.4–16.7]	38	
Diarrhoea	Men	2 946	12.7	476	16.4	[15.1–17.8]	34	0.436
	Women	3 439	13.0	581	17.1	[15.9–18.5]	35	
Nausea	Men	1 887	8.1	391	21.1	[19.2–23.0]	25	0.522
	Women	4 369	16.5	873	20.4	[19.2–21.6]	29	
Swollen legs	Men	1 953	8.4	870	45.1	[42.8–47.3]	4	<0.001
	Women	4 103	15.5	1 354	33.5	[32.1–35.0]	10	
Difficulty in emptying the bladder	Men	3 365	14.5	995	29.9	[28.4–31.5]	17	<0.001
	Women	2 366	8.9	539	23.1	[21.4–24–9]	25	
Frequent urination	Men	2 597	11.2	738	28.8	[27.0–30.6]	18	<0.001
	Women	2 637	10.0	624	24.2	[22.5–25.9]	24	
Stress incontinence	Men	256	1.1	90	35.7	[29.8–42.0]	9	<0.001
	Women	4 541	17.2	762	17.0	[15.9–18.1]	36	
Erectile dysfunction^a^	Men	4 289	18.5	1 362	32.1	[30.7–33.5]	14	-
	-	-	-	-	-	-	-	
Pelvic pain^a^	-	-	-	-	-	-	-	-
	Women	3 963	15.0	1 008	25.8	[24.4–27.2]	20	
Shortness of breath	Men	1 912	8.3	960	50.9	[48.6–53.2]	2	0.139
	Women	2 048	7.7	976	48.5	[46.3–50.7]	4	
Hoarseness	Men	1 677	7.2	293	17.7	[15.9–19.6]	30	0.147
	Women	2 105	8.0	405	19.6	[17.9–21.3]	32	
Urge incontinence	Men	1 184	5.1	322	27.7	[25.2–30.4]	20	0.102
	Women	1 896	7.2	468	25.0	[23.1–27.1]	23	
Loss of appetite	Men	1 359	5.8	256	19.2	[17.1–21.4]	27	0.767
	Women	1 720	6.5	330	19.6	[17.7–21.6]	31	
Blood in stool/rectal bleeding	Men	1 103	4.7	366	33.7	[30.9–36.6]	12	0.963
	Women	1 182	4.5	392	33.8	[31.1–36.6]	9	
Pelvic pain during intercourse^a^	-	-	-	-	-	-	-	-
	Women	2 091	7.9	552	26.6	[24.7–28.6]	18	
Fever	Men	841	3.6	211	25.3	[22.4–28.4]	22	0.18
	Women	1 111	4.2	306	28.0	[25.4–30.8]	16	
Difficulty swallowing	Men	781	3.4	254	33.2	[29.9–36.7]	13	0.205
	Women	946	3.6	332	36.2	[33.1–39.4]	7	
Weight loss	Men	768	3.3	185	24.8	[21.7–28.1]	23	0.758
	Women	722	2.7	178	25.5	[22.3–28.9]	22	
Incontinence without stress/urge	Men	328	1.4	111	34.7	[29.5–40.2]	11	0.703
	Women	830	3.1	272	33.5	[30.3–36.9]	11	
Pain/burning when urinating	Men	384	1.5	156	41.4	[36.4–46.5]	6	0.002
	Women	662	2.5	333	51.5	[47.5–55.4]	3	
Lump/swollen lymph nodes	Men	268	1.2	109	40.8	[34.9–47.0]	7	0.784
	Women	543	2.1	223	41.8	[37.6–46.2]	5	
Black stool	Men	451	1.9	68	15.4	[12.1–19.1]	35	0.093
	Women	328	1.2	64	20.1	[15.8–24.9]	30	
Repeated vomiting	Men	243	1.0	62	26.8	[21.2–33.0]	21	0.006
	Women	400	1.5	146	37.6	[32.8–42.7]	6	
Vaginal bleeding after intercourse^a^	-	-	-	-	-	-	-	-
	Women	612	2.3	187	30.9	[27.2–34.7]	13	
Postmenopausal bleeding^a^	-	-	-	-	-	-	-	-
	Women	370	1.4	118	33.1	[28.2–38.2]	12	
Blood in urine	Men	125	0.5	86	69.9	[61.0–77.9]	1	0.272
	Women	159	0.6	116	75.8	[68.2–82.4]	1	
Blood in semen^a^	Men	94	0.4	45	48.9	[38.3–59.6]	3	-
	-	-	-	-	-	-	-	
Coughing up blood	Men	42	0.2	18	43.9	[28.5–60.3]	5	0.415
	Women	20	0.1	11	55.0	[31.5–76.9]	2	
Blood in vomit	Men	32	0.1	11	39.3	[21.5–59.4]	8	0.683
	Women	22	0.1	6	33.3	[13.3–59.0]	17	

*Differences in GP-contacts with a symptom between genders were tested using chi-square tests

^a^ *Total numbers for each gender specific symptoms may not add up to full sample, due to the answer “do not wish to answer” was considered as missing (1.1*–*4.6* %*) in the analyses*

In total, 37 % contacted the GP with at least one symptom. For almost 2/3 of the reported symptoms, no statistically significant differences in reporting contacts to GP were found between the genders. However, women more often than men contacted the GP with repeated vomiting, coughing, tiredness and lack of energy, whereas men more often than women contacted the GP with stress incontinence, difficulties emptying the bladder, frequent urination, night-time urination and swollen legs (Table 3).

### Summary of main findings

This population based nationwide study demonstrated that symptoms were common; about 9 out of 10 individuals reported at least one symptom within the preceding four weeks. On average, women reported more symptoms than men; however, for some symptom the prevalence was higher for men. The majority of reported symptoms were not presented to the GP; the proportion of respondents contacting the GP with at least one symptom was 37 %. For 2/3 of the reported symptoms no gender differences in GP contacts were found.

### Strengths and limitations of the study

This study is a large nationwide population based study, including 100,000 individuals randomly selected from the Danish CRS register, representative of the adult Danish population aged 20 or above. To our knowledge such a large-scale population based study, investigating a wide range of self-reported symptoms covering specific and nonspecific cancer alarm symptoms as well as frequently occurring symptoms, has not previously been conducted.

The response rate of 52.2 % was comparable or even higher compared to previous surveys measuring symptom prevalences in the general population [27]. However, it is unknown whether individuals who had experienced symptoms might have been less or more inclined to participate in the study.

Information on symptoms and healthcare seeking decisions was self-reported, and respondents were asked to recall which of the 44 symptoms they had experienced in the preceding four weeks, and whether they at any time had contacted the GP with the symptoms they had experienced within the past four weeks. However, recall bias cannot be ruled out in questionnaire studies [28]. Some may misplace previous experiences of symptoms into the specified timeframe due to the severity of the symptoms or because they had contacted the GP about them [29]. Others may have forgotten about the experience of symptoms or GP contact because the symptom turned out to be inconsequential, or simply due to memory decay [30]. A higher proportion of individuals reporting GP contact with a symptom was found compared to other studies which might be explained by the unspecified timeframe for GP contact. In particular, this may be the case for the more frequently occurring symptoms such as back pain.

The web-based questionnaire was not available in a paper version, which might have prevented some individuals from participating in the study, especially the elderly. However, this possible selection bias was sought minimised by offering individuals without a computer the possibility to complete the survey by telephone interview.

### Symptoms and measurement – The Symptom Iceberg

When measuring symptoms, it is essential to define what a symptom is and how to measure it. As stated by Kroenke ’symptoms research is a fertile field’ [31], and we need to be more explicit about the way we conceptualise and measure symptoms. In this study we consider symptoms to be subjective interpretations of sensations and bodily changes, which are not necessarily an indication of an underlying disease.

Since no gold standard for measuring symptoms exits, studies on the prevalence of reported symptoms use different methodological approaches, which complicate comparison of the results between studies. However, despite the methodological differences, our results regarding the most frequently experienced symptoms are broadly consistent with previous symptom research [6, 8, 32]. This study focuses on individual reported symptoms, the total number of reported symptoms and corresponding contacts to the GP. Future studies might address how specific clusters of symptoms may affect the proportion of GP contacts.

### Gender differences

Some studies on symptoms and GP contacts suggest that men are less likely than women to report symptoms and to contact the GP [33]. However, other studies suggest that once a symptom is experienced and recognised, there are no gender differences in the tendency to contact the GP [5, 34, 35]. The results of this study show that for almost 2/3 of the reported symptoms, no statistically significant gender differences in reporting contact to GP were found.

### GP contacts - The “surfaced” part of The Symptom Iceberg

We found that 37 % contacted the GP with at least one of the symptoms experienced within the preceding four weeks. This proportion is relatively high compared to existing literature [5, 11–13, 27]. The original concept about “The Symptom Iceberg” was that approximately 10 % of all symptoms resulted in contact to the GP [36]. Our proportion of self-reported GP contacts might be higher as a result of the wording of the questions, different methodological approaches, or because of the changed cultural differences in the arena where people and GPs meet. Current medical practice is characterised by a focus on risk reduction and early detection of illness, which combined with developments in biomedical knowledge and diagnostic technologies has expanded “the pool of potential symptoms” [37]. Thus, more bodily changes, feelings or sensations may be designated as potential signs of disease. It is therefore to be expected that the pool of self-reported symptoms increases, and we may see a higher frequency of healthcare seeking. However, this should be further explored.

The decision on whether to contact a GP is based on a complex mix of physical, psychological and social factors [38]. The same symptom may by some people be regarded as harmless, while others may consider it as being too serious to ignore. The persistence of a symptom may also influence the interpretation of the symptom. These considerations or interpretations of the symptom will affect the decision on whether or not to contact the GP. The key issue seems not always to be the symptom itself.

Early diagnosis and prompt treatment are generally presumed to be a key to a better prognosis of most illnesses. An enhanced understanding of healthcare-seeking behaviours may assist health care professionals in identifying patients who are at risk of postponing contact to the GP and may help development of health campaigns targeting these individuals.

The literature indicates that multiple factors may affect peoples’ decision to seek healthcare. In this study we focused on prevalence and gender differences with regard to reporting of symptoms and contact to the GP. Future studies should explore other possible factors, which might trigger the individual to contact the GP, including age, characteristics of the symptoms and sociocultural factors such as use of social network in relation to a symptom.

## Conclusions

This study provides a comprehensive overview of the prevalences of 44 different self-reported symptoms and the corresponding proportions of GP contacts in a large nationwide population based study. More than 9 out of 10 individuals reported having experienced at least one symptom and 37 % had contacted the GP with a symptom. For almost 2/3 of the reported symptoms no gender differences were found concerning the proportion leading to GP contacts.
